# Healthcare Services for Undocumented Migrants: Organisation and Costs from the Italian NHS Perspective

**DOI:** 10.3390/ijerph192416447

**Published:** 2022-12-08

**Authors:** Elisabetta Listorti, Aleksandra Torbica, Silvano G. Cella, Gianfrancesco Fiorini, Giovanni Corrao, Matteo Franchi

**Affiliations:** 1 Centre for Healthcare and Social Care Management (CERGAS), SDA Bocconi, 20136 Milan, Italy; 2 Laboratory of Pharmacology and Pharmacoepidemiology, Department of Clinical Sciences and Community Health, University of Milan, via Vanvitelli 32, 20129 Milan, Italy; 3 National Centre for Healthcare Research and Pharmacoepidemiology, University of Milano-Bicocca, 20126 Milan, Italy; 4 Unit of Biostatistics, Epidemiology and Public Health, Department of Statistics and Quantitative Methods, University of Milano-Bicocca, 20126 Milan, Italy

**Keywords:** undocumented migrants, migrants, adherence, diabetes, cost, expenditure, organisation

## Abstract

Based on the principle of health equity, the Italian National Health Service is known worldwide for being a universalistic system that guarantees healthcare services for all its population, among which there are undocumented migrants. A commitment for their health needs is further motivated by their lower utilisation rates of healthcare services, which becomes even more crucial when considering chronic conditions such as diabetes that require adherence and continuity of care. However, the need for more official data has resulted in little research documenting these healthcare usage patterns. For this reason, our objective has been to deepen, from the Italian NHS perspective, the quantity, costs, type, preventability and organisation of healthcare services directed to undocumented migrants. We used official healthcare data from the Lombardy Region, which enable the identification of people receiving the STP code (undocumented migrants) and of people with foreign citizenship (documented migrants). After quantifying the average annual amount and expenditure for healthcare services grouped by Italian citizens, documented migrants and undocumented migrants for all clinical conditions (quantity and costs), we performed three primary investigations where we enlightened differences between the three mentioned groups focusing on the diagnosis of diabetes: (i) mapping the types of healthcare services used and their characteristics (type); (ii) quantifying the impact of preventable hospital admissions (preventability); (iii) examining the healthcare patterns linking pharmaceutical prescriptions with hospital accesses (organisation). Our results reveal significant differences among the three groups, such as more urgent hospital admissions, more preventable complications, and a higher recurrence in terms of access and costs to hospital services rather than pharmaceutical prescriptions for undocumented migrants. These findings can represent the leverage to raise awareness toward the emerging challenges of the migrant health burden.

## 1. Introduction

The Italian National Healthcare Service is known worldwide for being a universalistic system that guarantees healthcare services for all its resident population, regardless of personal, ethnic, social or economic characteristics [1]. The universalistic approach is also valid for undocumented migrants, who have the right to urgent care, essential care, diagnosis of preventive care, and treatment of potentially dangerous infectious diseases [2]. The Legislative Decree 286/98 that clarifies this entitlement [3] made Italy the first in Europe to address migrants’ health and access to health services through specific policies [4]. The commitment to migrants’ health is further motivated by the widespread phenomenon of migration that concerns all countries worldwide, with the number of international migrants estimated to be almost 281 million globally in 2020 [5]. As for Italy, around 10% of the population is immigrant, 8% of whom are undocumented [6]. Inclusive health care policies that expand access to health services for undocumented migrants have been documented to lead to better outcomes for undocumented migrants [7], but they are still not diffused in all European countries [8,9]. Moreover, within countries where these policies are put in place, migrants are shown to have a low utilisation rate of healthcare services [10,11,12,13], although higher for urgent conditions (documented by urgent hospitalisations or admissions to the emergency department, for instance) [14], which raises the concern for the absence of continuity of care [15].

An example of chronic pathology that urges continuity of care is diabetes. In recent years, diabetes has received greater attention due to the increase in prevalence and the importance of prevention and follow-up. The last IDF Diabetes Atlas declared that 537 million people worldwide suffer from this pathology, resulting in 6.7 million deaths and a total health expenditure of at least 966 billion dollars [16]. In Italy, it has been estimated that the disease annually burdens the National Healthcare Service (NHS) with EUR 9 billion of direct costs, with the average annual cost of care for a single diabetic patient accounting for EUR 2756 [17,18]. Moreover, the lack of therapeutical adherence can cause several complications and costly preventable hospitalisations [18,19]. Notwithstanding some differences that can occur between territorial contexts, the Italian literature reports that around 50% of costs of healthcare services are due to hospitalisation episodes, followed by around 35% of costs for pharmacological treatments and the remainder for specialistic services [17,18,20]. This distribution holds when patients receive appropriate treatments and are followed over time, which is not always the case for undocumented migrants. Several factors undermine their access to healthcare services, in fact, such as language barriers, fear of being reported to the police, poor language skills, lack of networks with native citizens, lack of knowledge about the healthcare system and lack of knowledge about informal networks of healthcare professionals [21,22,23,24]. An additional obstacle encountered by undocumented migrants in Italy is that they cannot access General Practitioners (GPs), whom the SSN contracts to provide primary care, preventive care and gatekeeping referrals to specialised care for all legal residents [25]. This limitation raises doubts about the care pathways that chronic undocumented migrants requiring essential care should follow. All the mentioned barriers may lead to alternative healthcare-seeking behaviours such as self-medication or delayed treatment, revealing different access to care for the migrant population that undermines the core public health concept of equity [26].

Multiple researchers have studied the phenomenon, focusing mainly on documented migrants [12,27,28,29]. The focus on documented migrants is motivated by the lack of official data about undocumented migrants [30], which are more often drawn from interviews, ad hoc studies, or collected from charitable organisations providing healthcare services [10,22,31,32,33]. Within the Italian context, undocumented migrants receive a temporary code called STP (STP for “straniero temporaneamente presente”, i.e., “foreigner temporarily present”), which expires after six months. The documentation can be renewed, but often the code is changed. The change in the identification code precludes studying the same individual over time using official data registries.

Given this complex picture, our objective has been to deepen from the NHS perspective the quantity, costs, type, preventability and organisation of healthcare services directed to undocumented migrants with diabetes. The choice of diabetes among the chronic conditions was motivated by its relevant impact in terms of prevalence and costs, together with the presence of interesting features such as its related usage of multiple types of healthcare services (e.g., not only concentrated on pharmaceutical prescriptions) and the possibility of its clear identification through the diagnosis information contained in all types of administrative data (differently, for example, from mental illness that is often difficult to track through hospital admissions because it is less reported).

Our work is built on the usage of official healthcare data from the Lombardy Region, which enables the identification of people receiving the STP code (undocumented migrants) as well as of people with foreign citizenship (documented migrants). As a starting point, we initially quantify the average annual amount and expenditure for healthcare services grouped by Italian citizens, documented migrants and undocumented migrants for all clinical conditions (quantity and costs). We then performed three main investigations where we enlighten differences between the three mentioned groups: (i) mapping the types of healthcare services exploited and their characteristics (type); (ii) quantifying the impact of preventable hospital admissions (preventability); (iii) examining the healthcare patterns linking pharmaceutical prescriptions with hospital accesses (organisation). We ultimately aim to answer the need to address the emerging challenges of the migrant health burden, as strongly advocated recently [34].

## 2. Materials and Methods

### 2.1. Data

Data were retrieved from the regional healthcare utilisation databases of the Lombardy Region in Italy, the most populated region in Northern Italy, accounting for about 10 million inhabitants (17% of the national population). The database collects a variety of information on residents who receive NHS assistance (NHS beneficiaries), among which are (i) diagnoses and procedures of inpatients of public or private hospitals (coded according to the ICD-9-CM codes), (ii) outpatient drug prescriptions (coded according to the Anatomical Therapeutic Chemical (ATC) Classification System), (iii) specialist visits and (iv) access to emergency departments (ED), all reimbursable by the NHS. For each healthcare service provided, the dataset contains information about its cost. Additionally, the dataset contains some personal data, including the ownership of an STP code. The time window of the study is 2018-2019 due to the availability of the STP information and the exclusion of the COVID-19 period.

As a unique personal identification code is used for all regional databases, it is possible to exploit a record linkage to search out the complete care pathway of beneficiaries of the NHS. However, within this study, we were not able to perform a longitudinal analysis, since STP codes are re-assigned every six months, and thus we are not able to follow undocumented migrants for more than such a time interval (of which we do not know the official beginning and end).

In order to preserve privacy, identification codes were automatically converted into anonymous codes, and the inverse process is prevented by deleting the conversion table. Since all the analyses are based on anonymised data, no permission from Ethic Committees is necessary to collect and analyse these data.

### 2.2. Methods

As for the baseline snapshot on the amount and expenditure for healthcare services related to all clinical conditions, we calculated both the number of services provided and their costs grouped by Italian citizens, documented migrants and undocumented migrants for four types of service: hospital admissions, pharmaceutical prescriptions, specialistic visits and accesses to the emergency department [quantity, costs]. Moreover, we also calculated the number and percentages of patients requiring healthcare services grouped by age and sex, for each subpopulation.

### 2.3. Mapping the Types of Healthcare Services Used and Their Characteristics [Type]

As for the first analysis, we investigated the magnitude of different sources of health expenditures strictly related to diabetic diagnosis.

We decided to adopt the NHS perspective to state at an aggregated level how much the distribution of the costs of the same clinical condition can vary depending on the migration status. More specifically, we selected: (i) hospital admissions containing within the diagnosis the ICD-9 cm diagnostic code 250; (ii) pharmaceutical prescriptions with the ATC drug code A10; (iii) specialistic visits with diabetologists using regional codes 89018 or 897A8; (iv) access to the emergency department containing within the diagnosis the ICD-9 cm diagnostic code 250. Our main output is a graph of the different distributions of these expenditures, to which we added a graph of the distribution of the number of accesses to the same services. We then calculated some parameters for the hospital services (i.e., length of stay and elective/urgent), and checked if there were statistically significant differences among the groups of patients by using the Kruskal–Wallis test for the length of stay and the Chi-square test for type of hospitalisation.

### 2.4. Quantifying the Preventable Hospital Admissions [Preventability]

We identified among the hospital admissions the ones referring to diabetic avoidable complications. Using the same ICD codes used by PNE National Healthcare Outcomes Programme [35], we distinguished amputation, short-term complications, long-term complications, and non-controlled diabetes. We then calculated the percentage of admissions costs related to each of these conditions and checked if there were statistically significant differences among the groups of patients by using the Chi-square test.

### 2.5. Examining the Healthcare Patterns Linking Pharmaceutical Prescriptions with Hospital Accesses [Organisation]

We calculated the percentage of pharmaceutical prescriptions which occurred within 10 days from the discharge of a hospital admission and checked if there were statistically significant differences among the groups of patients. The rationale of this focus is motivated by the possible higher recurrence of prescriptions after hospital admissions for undocumented migrants, who otherwise have difficulty in finding other channels to receive drugs, as reported by anecdotal evidence.

## 3. Results

As a first step, descriptive statistics from the general registry are reported to characterise in terms of age and sex the three populations, i.e., Italian citizens, documented migrants and undocumented migrants (Table 1). Data should be read knowing the limitation of the STP code, which may preclude from identifying univocally individuals that are present within the registry twice with two different codes.

We can notice relevant differences, especially in the age distribution. Undocumented and documented migrants requiring healthcare services are not often older than 70 (only 11% and 3% of services, respectively, for documented and undocumented migrants), different from the 44% of services in the case of Italian citizens. More precisely, services for undocumented migrants are most often dedicated to young people: 61% of the services are provided to people aged less than 30 years, well above the 7% and 10% in case of Italian citizens and documented migrants.

Table 2 reports both the total number of accesses and the costs related to the healthcare services provided in 2018 and 2019, which help to gain a picture of the distribution of accesses and costs among the different populations.

We highlight two aspects of interest: (i) the percentage of services covered by undocumented migrants out of the total services provided varies greatly depending on the service considered, going from 0.01% for pharmaceutical prescriptions up to 0.13% for hospital admissions, thus revealing a different usage of the types of healthcare services compared to Italian citizens and documented migrants; (ii) the average percentage of accesses made by undocumented migrants across all the healthcare services is 0.07%, which raises attention given the composition of the population in Italy, where around 0.8% are undocumented migrants.

**Figure 1:**
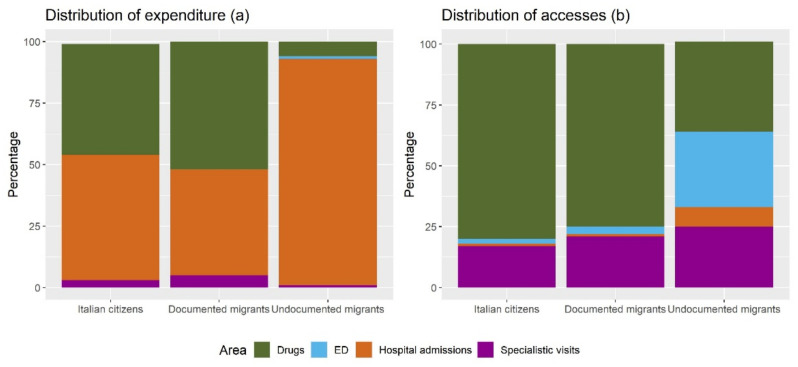
Distribution of expenditure (**a**) and accesses (**b**) for different healthcare services, grouped by population.

As for the objective of mapping the types of healthcare services exploited and their characteristics, our main output is a graph of the different distributions of the different expenditures (i.e., hospitalisations, pharmaceuticals, specialistic visits, access to ED), which is shown in Figure 1a. While the percentage of costs due to hospital admissions for Italian patients reflects the statistics reported in the literature [18], and it is slightly less for documented migrants, the same does not happen for undocumented migrants. The percentage of healthcare expenditure dedicated to hospital admission, in fact, is 51% for Italian citizens and 43% for documented migrants, while it raises to 92% for undocumented migrants. This difference in the expenditure for hospital admissions mirrors the difference occurring in the pharmaceutical expenditure. In fact, the pharmaceutical expenditure accounts for 6% of the total expenditure of undocumented migrants, much less compared to the 45% and 52% of the total expenditure of Italian citizens and documented migrants, respectively.

To explain the existing differences in the distribution of costs, we also examined the distribution of accesses to each of the healthcare services, which is reported in Figure 1b. From the graph we can see that, out of the overall accesses to the mentioned healthcare services, both for Italian citizens and documented migrants, the greatest part (accounting for 80% and 75%) is dedicated to pharmaceutical prescriptions. For undocumented migrants, instead, this share is strongly reduced (37%), in favour of a greater amount of accesses to the Emergency Department. Additionally, the percentage of accesses to hospital, which is 1% and 1% for Italian citizens and documented migrants, raises to 8% for undocumented migrants.

Focusing on the hospital admissions, we examined both the length of stay and the classification of elective/urgent episodes. While the percentage of urgent admissions is 56% for Italian citizens and 63% for documented migrants, it raises to 81% for undocumented migrants (*p* < 0.0001). The length of stay is 13.7 days for Italian citizens, 13.1 days for documented migrants, and 14.3 days for undocumented migrants (*p* < 0.0001).

**Figure 2:**
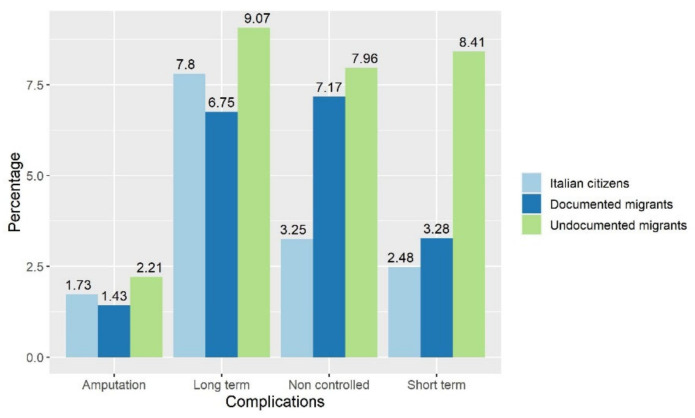
Percentage of preventable hospital admissions costs, by type and by population of patients.

As for the objective of quantifying the preventable hospital admissions, Figure 2 shows the percentage of the hospital costs due to amputations, short term complications, long term complications and non-controlled diabetes. For all these indicators, undocumented migrants show higher percentages, and the *p*-values confirm the significance of this result for long-term complications (*p* = 0.0029), short-term complications (*p* < 0.001) and non-controlled diabetes (*p* < 0.001), while no significant differences emerge in the case of amputations (*p* = 0.111).

As for the linkage between pharmaceutical prescriptions and accesses to hospitals, 7.4% of Italian patients have accessed a pharmacy for a pharmaceutical prescription within 10 days of a hospital discharge. However, this percentage increases to 8.4% for documented migrants and to 10.1% for undocumented migrants (*p*-value < 0.0001).

## 4. Discussion

The latest literature has proved undocumented migrants’ problems accessing healthcare services, thus receiving inadequate or insufficient care [13]. This finding represents a threat to one of the main principles driving health policies, that is, equity, and for this reason calls for further research dedicated to understanding the mechanisms that can be improved/eradicated to change the situation. However, the need to monitor the quantity and quality of services provided collides with the lack of data [30] and the difficulty of following a rapidly moving population over time. The temporary code assigned to undocumented migrants in Italy represents an additional challenge to the activity of tracking individual care pathways over time.

Our study tries to handle these limitations and map, from the NHS perspective, the quantity, costs, type, preventability, and organisation of healthcare services directed to diabetic undocumented migrants. First, general descriptive statistics on hospital admissions show that the majority of undocumented migrants recurring to this healthcare service are below the age of 30 years, which contrasts with that of hospital patients who are Italian citizens and documented migrants, who belong to older age categories. When focusing on diabetes diagnosis, considering the overall access to healthcare services, undocumented migrants make more use of emergency department and hospital admissions than pharmaceutical prescriptions and specialistic visits. Regarding the expenditures for hospital admissions, undocumented migrants show higher percentages of avoidable hospitalisations, such as amputations, short and long-term complications, and non-controlled diabetes. Moreover, the percentage of patients receiving pharmaceutical prescriptions right after a hospital discharge is higher for undocumented migrants.

Our results reveal a discrepancy among the treatments reserved for Italian citizens, documented migrants, and undocumented migrants, confirming the patterns documented by the literature, i.e., more use of the emergency department and hospitalisations by migrants [15]. The large gap between entitlements and utilisation of healthcare services reported by this study and the great majority of previous studies in the field [13] echoes the WHO call for improving health for all and reducing health inequalities by addressing the challenges faced by migrants [36]. As a starting point, a reflection is needed on the barriers encountered by migrants in accessing all the healthcare services and the actions undertaken to overcome them.

One of the barriers consists of a lack of knowledge about the entitlements owned by undocumented migrants [10], which calls for the need to develop information and communication systems to improve access to healthcare for immigrants and integrate immigrants within the healthcare system. Hence, national policies addressing the need to improve health education (such as the 2001–2003 plan that stated that local health offices should promote information campaigns for migrants, or the 2006–2008 plan that aimed to promote education programmes in cooperation with volunteer and not-for-profit organisations), should be pursued and fostered [4].

However, after informing undocumented migrants about the services they are entitled to, attention should be paid to the organisation that enables them to avoid ‘implementation gaps’ [8]. A significant example is the inconsistency between the right of diabetic undocumented migrants to be treated as needing essential care, and the impossibility of accessing GPs and thus receive drug prescriptions. We suggest that the higher use of pharmaceutical prescriptions right after a hospital discharge may reveal a difficulty in getting pharmaceutical prescriptions through other channels.

Beyond all this, the relevant role played by informal care channels in providing healthcare services to patients has to be mentioned. Charitable organisations and non-governmental organisations often provide health care services at prevention, treatment, and rehabilitation levels, and are diffused through the Italian territory [31,37,38]. Their essential role in the healthcare services has beneficial effects, such as a higher utilisation of health care services by migrants thanks to the implementation of a series of migrant-oriented practices [24]. However, the absence of integration between the NHS and these organisations causes the impossibility of drawing a complete picture of the use of healthcare services by undocumented migrants, leaving open several questions. How many healthcare services are provided by informal channels and thus are not monitored and eventually neglected by the NHS? How many undocumented migrants receive services from both formal and informal channels, with the risk of duplications or inappropriateness?

One limitation of our research is the exclusion from the study period of the year 2020, and thus the consideration of the SARS-CoV-2 pandemic. One work has deepened the effects of the pandemic on the follow-up and pharmacological treatment of chronic diseases and found that in undocumented migrants the number of consultations decreased at the beginning of 2020 [39]. For this reason, future research may extend our analysis to 2020 to further support the design of new strategies to better assist the very poor during epidemics.

Future research might also widen the analysis to other chronic conditions which also require adherence and continuity of care, to investigate if different patterns are emerging between clinical areas in terms of access to services and type of usage.

To the best of our knowledge, our study is the first to investigate the different healthcare services related to diabetes from an NHS perspective focusing specifically on undocumented migrants, and the first to quantify the related expenditure. This focus poses several challenges, which all originate from using the STP code. In fact, despite the fact that the STP code remains the only code that identifies undocumented migrants within Italian official healthcare data, it precludes from identifying univocally a person due to the multitude of codes that the same person may receive over time, and the lack of a dataset keeping track of this process. The main limitation that emerges as a consequence of using the STP code is the impossibility of observing individuals over time. As such, we could not follow up on patients and track the individual health trajectory, e.g., the diagnosis and treatment of the same illness, the presence of multiple illnesses, the evolution of the health status over time, etc. When concentrating on diabetes, we could only consider healthcare services at an aggregated level. The use of aggregated data prevented us from drawing causal claims and specific conclusions on the individual care pathways. Examples of in-depth analysis that did not have these limitations can be found in two previous Italian works that quantified the economic burden of diabetes [20,40] by identifying individuals with diabetes (not considering undocumented migrants) and tracking their health expenditures over time. This approach also enabled the authors to track the healthcare services not strictly related to diabetes, but the comorbidities and complications correlated with it. It would be useful to produce such results for undocumented migrants as well, since policy makers would benefit from them when designing new policies directed to this population. Future efforts should be spent by the whole scientific community in reinforcing the advocacy process for obtaining central decision makers to address this data issue.

However, we believe that our research enriches the literature on undocumented migrants by enlarging the scope of research beyond hospital admissions, which have been the main focus of previous studies [25,29]. The consideration of multiple types of healthcare services enables the drawing of a more complete picture of the phenomenon. Above all, even if at an aggregated level, we believe that documenting the magnitude of the healthcare supply to migrants is the first step needed to address the emerging challenges of the migrant health burden.

## 5. Conclusions

Within this study, we aimed to provide enlightenment on the actual healthcare services provision directed to undocumented migrants, compared to documented migrants and Italian citizens. By building on the richness of the available dataset, we characterised healthcare usage within several aspects: quantity, costs, type, preventability, and organisation. Results reveal different patterns for undocumented migrants, such as more urgent hospital admissions, more preventable complications, and a higher recurrence in terms of access and costs to hospital services rather than drugs. While these findings go in the same direction as previous studies, the quantitative analysis allowed by administrative data further supports the design of future actions that need to be implemented to reduce the reported differences. All in all, our study can represent the leverage to raise awareness toward the emerging challenges of the migrant health burden.

## Figures and Tables

**Table 1:** Number of patients receiving healthcare services provided in 2018-2019, grouped by sex and age class.

	All (No. of Patients)	Sex		Age Class			
		Female	Male	<30	30–49	50–69	70+
**Italian citizens**	918,936	524,360 (57%)	394,576 (43%)	67,204 (7%)	112,728 (12%)	334,765 (36%)	404,239 (44%)
**Documented migrants**	118,854	77,481 (65%)	41373 (35%)	12,119 (10%)	45,356 (38%)	48,409 (41%)	12,970 (11%)
**Undocumented migrants**	1747	476 (55%)	412 (45%)	1057 (61%)	329 (19%)	306 (18%)	55 (3%)

**Table 2:** Number of accesses and costs for different healthcare services, grouped by population.

Healthcare Service	Population	Number of Accesses	Total Cost
**Drug prescriptions**	Italian citizens	69,596,360	198,230,000
	Documented migrants	5,316,618	159,140,000
	Undocumented migrants	9172	357,989
**Hospital admissions**	Italian citizens	720,231	3,214,200,000
	Documented migrants	72,851	273,280,000
	Undocumented migrants	1069	5,216,012
**Specialistic visits**	Italian citizens	89,510,760	1,891,900,000
	Documented migrants	9,305,148	191,510,000
	Undocumented migrants	40,368	1,214,096
**Emergency department**	Italian citizens	15,051,754	164,080,000
	Documented migrants	1,698,704	18,297,606
	Undocumented migrants	14,971	146,886

## Data Availability

The data that support the findings of this study are available from Lombardy Region, but restrictions apply to the availability of these data, which were used under license for the current study, and so are not publicly available. Data are, however, available from the Lombardy Region upon reasonable request.
